# The ethanolic extract of *Osmanthus fragrans* var. *thunbergii* flowers ameliorates depressive‐like behaviors of mice by modulating the serotonin system and suppressing neuroinflammation

**DOI:** 10.1002/fsn3.4270

**Published:** 2024-06-12

**Authors:** Lu‐yao Luo, Rui Xue, Ting‐ge Wang, Jing‐wen Zhang, Shuo Li, Jin‐cao Li, Qiong‐yin Fan, Hua‐jin Dong, Yang Zhang, You‐zhi Zhang

**Affiliations:** ^1^ School of Pharmacy North China University of Science and Technology Tangshan China; ^2^ State Key Laboratory of Toxicology and Medical Countermeasures, Beijing Key Laboratory of Neuropsychopharmacology Beijing Institute of Pharmacology and Toxicology Beijing China; ^3^ Nanjing University of Chinese Medicine Nanjing China

**Keywords:** antidepressant, neuroinflammation, *Osmanthus fragrans* var. *thunbergii* flower, serotonin system, UPLC‐Q‐TOF‐HRMS

## Abstract

It is crucial to explore the impact of dietary interventions on depression and develop functional antidepressant foods, due to the significant side effects and poor treatment compliance of antidepressant drugs. *Osmanthus fragrans* flowers are edible and medicinal materials owing to their delightful floral aroma and significant health benefits. Here, we evaluated the antidepressant effects of the ethanolic extract of *O. fragrans* var. *thunbergii* flowers (OFE) and investigated the mechanisms of action on the serotonin system and neuroinflammation, and analyzed the main chemical components of OFE. A single administration of OFE significantly reduced the immobility duration in the forced swimming and tail suspension tests in mice without affecting locomotor activity. OFE exhibited selective enhancing effects on 5‐HTergic function in vivo, as demonstrated by its potentiating effects on 5‐hydroxytryptophan (5‐HTP)‐induced head‐twitch behavior and alleviation effects on reserpine‐induced ptosis deficits. In addition, OFE increased 5‐hydroxytryptamine (5‐HT) concentration and upregulated 5‐HT_1A_ expression in reserpine‐treated mice, further validating its effects on 5‐HT transmission. Interestingly, OFE significantly alleviated microglia activation and the production of inflammatory mediators, both in reserpine‐treated mice in vivo and lipopolysaccharide (LPS)‐stimulated BV‐2 cells in vitro. Additionally, 62 components in OFE were identified using ultra‐performance liquid chromatography quadrupole time‐of‐flight high‐resolution mass spectrometry, and glycoside derivatives were shown to be the major constituents of OFE. The present study showed that OFE can alleviate depressive‐like behaviors by modulating the serotonin system and reducing neuroinflammation. These results suggest that OFE can be valuable dietary supplements with therapeutic effects on depression.

## Graphical abstract

A single administration of Osmanthus fragrans var. thunbergii flowers ethanolic extract (OFE) exhibited antidepressant‐like effects in behavioral despair models on mice. OFE exhibited antidepressant‐like effects by modulating 5‐HT and suppressing neuroinflammation. Notably, OFE significantly alleviated microglia activation and the production of inflammatory mediators, both in reserpine‐treated mice in vivo and in LPS‐stimulated BV‐2 cells.

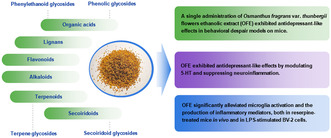

## 1 INTRODUCTION

Depression is a widely occurring mental disease that severely affects the daily lives of individuals, even resulting in suicidal tendencies (Smith, 2014). From the beginning of the 21st century, the incidence of depression has been steadily increasing. According to the World Health Organization (WHO), >350 million individuals worldwide suffer from depression, which has become the second largest number for disease worldwide and is projected to become the largest by 2030 (Ramadan & Mansour, 2020). Moreover, the number of individuals with depression is predicted to increase significantly in the post‐coronavirus of 2019 (COVID‐19) era and approximately one‐third of individuals who have survived COVID‐19 continue to experience persistent neurocognitive impairments (Santomauro et al., 2021). The commonly prescribed clinical antidepressants, such as selective serotonin reuptake inhibitors (SSRIs), serotonin and norepinephrine reuptake inhibitors (SNRIs), noradrenergic and specific serotonergic antidepressants (NaSSAs), and tricyclic antidepressants (TCAs), are primarily chemically synthetic compounds (Sarko, 2000). However, owing to the significant side effects and poor treatment compliance of synthetic antidepressants, there is an urgent need to develop complementary and alternative therapies from natural dietary supplements. Among the various complementary therapies, phytotherapy, which is based on herbal dietary supplements, have been recognized as a promising strategy for the prevention of depression (Hossen et al., 2021; Manosso et al., 2013; Wu,  Liu, Zhang et al., 2022; Zhang et al., 2023). A noteworthy example is *Hypericum perforatum* (Hypericaceae), commonly known as St. John's Wort, an herb that is widely prescribed for depression in Europe and also used as a dietary supplement in the United States (McFadden & Hooker, 2019).

*Osmanthus fragrans*, also known as sweet *Osmanthus*, or sweet olive, is a member of the Oleaceae family that is widely cultivated in southern and central China (Yao et al., 2020). The varieties of *O. fragrans* are categorized into four primary groups based on their morphological characteristics, among which *O. fragrans* var. *thunbergii* is the most extensively cultivated (Zhou, Zhao et al., 2017). *O. fragrans* flowers are utilized extensively as food ingredients in the preparation of tea, scented jams, wines, and various other products, owing to their delightful floral aroma and significant health benefits (Wu, Liu, Huang et al., 2022). Furthermore, *O. fragrans* flowers are edible medicinal materials, and have been used as a traditional Chinese medicine (TCM) to treat a wide range of diseases and conditions related to inflammation, including stomachache, toothache, asthma, rheumatism, and hepatitis (Wang et al., 2022).

A previous study has reported that chronic administration of *O. fragrans* fruit extract showed significant antidepressant‐like effects in the forced swimming tests (FST) and tail suspension tests (TST) in mice (Jawaid et al., 2015). Moreover, Hung et al. showed that chronic administration of *O. fragrans* flower extract improved depression‐like behavior in maternally deprived (MD) rats by enhancing the antioxidant capacity in the depression‐related brain regions of these rats (Hung et al., 2012). However, whether *O. fragrans* var. *thunbergii* flowers (OFE) consumed as a dietary supplement will have therapeutic effects on depression is unknown, and the potential mechanisms involved remain to be fully elucidated. Therefore, in the present study, we aimed to evaluate the antidepressant‐like effects of the OFE ethanolic extract and investigate its mechanisms of actions on the serotonin system and neuroinflammation, as well as illustrate the primary chemical components of OFE.

## 2 MATERIALS AND METHODS

### 2.1 Materials

The flowers of *O. fragrans* var. *thunbergii* were purchased form Meijiawu Co., LTD. (Quanzhou, China), and was identified by Professor Shushan Du from Beijing Key Laboratory of TCM Protection and Utilization, Beijing Normal University. A voucher specimen (JG‐2021‐12) was deposited in the Laboratory of Phytochemistry, Beijing Institute of Pharmacology and Toxicology.

Duloxetine (DLX) was purchased from Zhejiang Liaoyuan Pharmaceutical Co., LTD (Taizhou, China). Lipopolysaccharide (LPS, #L2630), 5‐hydroxytryptophan (5‐HTP, #H9772), yohimbine hydrochloride (#Y0002376) and reserpine (#83580) were purchased from Sigma‐Aldrich (San Francisco, United States). Antibody for 5‐HT_1A_ (#ab85615) was purchased from Abcam (Massachusetts, United States). The primary antibodies detected for ionized calcium binding adapter molecule 1 (Iba‐1, #17198 s) and nuclear factor kappa‐B (NF‐κB, #8242 s) were purchased from Cell Signaling Technology (Massachusetts, United States). Beta‐actin monoclonal antibody (#CW0096M) was purchased from Cowin Biotech Co., Ltd (Beijing, China). Goat anti‐mouse IgG antibody (#ZB‐2305) and goat anti‐rabbit IgG antibody (#ZB‐2301) were obtained from ZSGB Biotech Co., Ltd (Beijing China). Mouse ELISA kits for interleukin‐6 (IL‐6, #JL20268) and interleukin‐1β (IL‐1β, #JL18442) assay were purchased from J&L Biological Industrial Co., LTD (Shanghai, China). Mouse ELISA kit for TNF‐α (tumor necrosis factor‐alpha, # KE10002) assay was purchased form Proteintech Group, Inc (Rosemont, United States). Cellular NO (nitric oxide) assay kit (#S0021S) was purchased from Beyotime Biotechnology (Shanghai, China).

### 2.2 Preparation of OFE

The dried flowers of *O. fragrans* var. *thunbergii* (520 g) were refluxed and extracted for 2 h with a 10‐fold amount of 70% aqueous ethanol (v/v) three times. The collected extract was combined, filtered, and the filtrates were concentrated in vacuo under 50°C and then freeze‐dried to obtain extract powder (198.6 g, 38.19% w/w). The extract powder was stored at −20°C.

### 2.3 Chemical component analysis of OFE

The OFE was dissolved in 50% methanol aqueous solution to prepare the standard solution of 0.037 mg/mL concentrations. After filtration with a 0.22 *μ*m membrane, the chemical constituents of OFE were analyzed on a Waters H‐Class UPLC system equipped with an AB Sciex TripleTOF 4600 mass spectrometer. Chromatographic separation was achieved using a Waters ACQUITIY UPLC HSS T3 column (2.1 mm × 150 mm, 1.8 *μ*m), with column temperature maintained at 30°C. The mobile phases consisted of acetonitrile (A) and 0.1% formic acid water (B) and using a gradient elution. Elution conditions were as follow: 0–3 min 100% B; 3–8 min 100%–90% B; 8–16 min 90% B; 16–30 min 90%–82% B; 30–45 min 82%–75% B; 45–53 min 75%–60% B; 53–59 min 60%–10% B; 59–62 min 10% B and other 3 min to re‐equilibrate the column. The flow rate was 0.3 mL/min, and the injection volume was 2 *μ*L. The electrospray ionization (ESI) source was used to collect data in both positive and negative ion modes. The source parameters were as follows: declustering potential, 100 V; source temperature, 500°C; air curtain gas, 35 psi; auxiliary air pressure, 50 psi; atomization gas pressure, 50 psi; scanning range 50–1700 *m/z*. Product ion scans were performed using an *m/z* range of 50–1250 amu, a collision energy of ±40 eV, and a collision energy spread of 20 eV.

### 2.4 Animals

Male ICR mice weighing 18–22 g were purchased from SPF Biotechnology Co., Ltd (Beijing, China). The animals were housed in standard experimental conditions: room temperature 21 ± 2°C, humidity 40%–60%, 12 h:12 h‐light/dark cycle (lights on at 8:00 a.m.). Food and water were available ad libitum. Animals were allowed to have a period of acclimation before experiment. In behavioral tests, to minimize circadian changes and subjective influence, animals in each group were intermixed during the observation (8:00–12:00 a.m.) and the observers were unaware of the treatment conditions. The animal experiments were performed in strict accordance with the national institutes of health guide for the care and use of laboratory animals.

### 2.5 Antidepressant‐like effects of OFE in the behavioral despair models in mice

#### 2.5.1 Drug treatment

Fifty mice were randomly divided into five groups (10 mice in each group): a vehicle group, a DLX group (20.0 mg/kg), and different doses of OFE groups (0.3, 1.0 and 3.0 g/kg). The doses of DLX and OFE selected were based on the results from previous studies and preliminary experiments (Hung et al., 2012; Xue et al., 2013). After a 7 days period of environmental adaptation, mice were orally administrated with DLX, OFE or distilled water 1 h prior to the FST, TST and LAT.

#### 2.5.2 FST

The experiment was performed with mice according to the protocol of Porsolt et al. (Porsolt et al., 1977) with minor modifications. Mice were individually placed in cylindrical containers (diameter 12 cm, height 20 cm, contained 10 cm of water maintained at 25 ± 1°C) for 6 min. The duration of immobility during the last 4 min was recorded by a blinded observer using a stopwatch. Mice were considered to be immobile when they floated motionless, with only movements necessary to maintain their heads above the water.

#### 2.5.3 TST

The TST procedure was performed as previously described with minor modifications (Steru et al., 1985; Zhang et al., 2007). Mice were suspended for 6 min on the top of the apparatus using adhesive tape placed approximately 2 cm from the tip of their tails, and the immobility time was measured for the last 4 min. Mice were considered to be immobile when they hung passively without struggling.

#### 2.5.4 Locomotor activity test (LAT)

Mice were placed individually in an activity test chamber (25 × 25 × 25 cm) equipped with Dig‐Behv video tracking system (Jiliang Software Technology Co., Ltd., Shanghai, China). The total distance of each mouse was automatically recorded during the 10‐min test. The enclosures were cleaned with 75% ethanol during the test interval.

### 2.6 Effects of OFE in the pharmacological models in mice

#### 2.6.1 5‐HTP induced head‐twitch test

To reveal whether the serotonergic system was involved in the antidepressant‐like effects of OFE, 5‐HTP induced head‐twitch test in mice was conducted (An et al., 2008). Fifty mice were randomly divided into five groups (10 mice in each group): a vehicle group, a DLX group (10.0 mg/kg), and different doses of OFE groups (0.3, 1.0, and 3.0 g/kg). Sixty minutes after oral administration of vehicle or drugs, all mice in each group were intraperitoneally injected with 5‐HTP (180.0 mg/kg). Then, 5 min later, mice were placed into a transparent box to record the number of head‐twitch within 15 min. Head‐twitch behavior was defined as rapid movements of the head.

#### 2.6.2 Yohimbine toxicity potentiation test in mice

To reveal whether the noradrenergic system was involved in the antidepressant‐like effects of OFE, yohimbine toxicity potentiation test in mice was performed (Lapin, 1980). Fifty mice were randomly divided into five groups (10 mice in each group): a vehicle group, a DLX group (20.0 mg/kg), and different doses of OFE groups (0.3, 1.0, and 3.0 g/kg). Sixty minutes after oral administration of vehicle or drugs, yohimbine (30.0 mg/kg) was subcutaneously injected, and then the number of dead mice was recorder in the following 24 h.

#### 2.6.3 Reserpine‐induced monoamine depletion model in mice

Reserpine‐induced monoamine depletion model in mice was developed according to the method described by Bourin et al. with some modifications (Bourin et al., 1983; Zhu et al., 2023). Fifty mice were randomly divided into five groups (10 mice in each group): vehicle group (0.1% acetic acid solution), model group (reserpine, 5.0 mg/kg), DLX group (reserpine 5.0 mg/kg + DLX 20.0 mg/kg), and OFE groups (reserpine 5.0 mg/kg + OFE 1.0 and 3.0 g/kg). After environmental adaption for 7 days, DLX and various doses of OFE were administered orally. Sixty minutes later, mice were intraperitoneal injected with reserpine at a dose of 5.0 mg/kg (dissolved in sterile, endotoxin‐free 0.1% acetic acid solution), except for vehicle group. Then, the antagonism effects of OFE and DLX against reserpine‐induced ptosis, akinesia and hypothermia were recorded and evaluated as follows.

##### Ptosis

Sixty minutes after the injection of reserpine, mice were placed on a shelf (20 cm above table), and the degree of ptosis was marked according to the following rating scale: 0, eyes open; 1, one‐quarter closed; 2, half closed; 3, three quarters closed; and 4, completely closed (Sánchez‐Mateo et al., 2007).

##### Hypothermia

Before drug administration, the rectal temperature of each mouse was measured using an electronic thermometer as basal temperature (T0,°C). Two hours after reserpine injection, the rectal temperature of each mouse was measured again (T1,°C). The difference (△T,°C) between T0 and T1 was calculated (△T = T0 − T1,°C).

##### Akinesia

Sixty minutes after the injection of reserpine, mice were placed in the center of a circle paper with a radius of 7.5 cm, and the number of akinesias were recorded. Mouse were deemed as akinesia when it was unable to walk out of the circle within 15 s.

After behavior testing, mice were sacrificed by cervical dislocation. The hippocampus samples were isolated on ice and used for monoamine assays, ELISA, and western blotting analysis.

### 2.7 The anti‐inflammatory effects of OFE in LPS‐stimulated BV‐2 cells

BV‐2, a murine microglial cell line, was cultured in Dulbecco's modified Eagle's medium (DMEM, Gibco) containing 10% fetal bovine serum (BioChannel Biotechnology Co., Ltd. Nanjing, China) in an incubator at 37°C with 5% CO_2_. We performed cell viability assays to select the appropriate dosage of OFE. The cell viability assay was performed following the instructions of the CCK‐8 (Cell Counting Kit‐8) kit (Yeasen Biotechnology Co., Ltd. Shanghai, China). The dose of LPS was determined by the pilot studies. Subsequently, we investigated the anti‐inflammatory effects of OFE in LPS‐stimulated BV‐2 cells. The experiment included seven groups: vehicle group, model group (LPS, 1.0 μg/mL), DLX group (LPS 1.0 μg/mL + DLX 10 μg/mL) and OFE groups (LPS 1.0 μg/mL + OFE 10, 30, 100, and 300 μg/mL). Cells were seeded in 24‐well plates (4 × 10^5^ cells/well) and cultured for 24 h. After pretreated with DLX and various dose of OFE for 1 h, cells were incubated with LPS (1.0 μg/mL) for an additional 24 h. The levels of TNF‐*α* and IL‐6 in the supernatant were determined using the ELISA kits, and the protein expression of Iba‐1 and NF‐κB was assayed using western blotting.

### 2.8 Antidepressant‐like effects of OFE in LPS‐induced depression model in mice

Mice were pretreated with LPS to induce depression‐like behaviors as previously described with minor modifications (Cazareth et al., 2014). Ninety mice were randomly divided into six groups (15 mice in each group): vehicle group (saline), model group (LPS 1.0 mg/kg), DLX group (LPS 1.0 mg/kg + DLX 20.0 mg/kg), OFE groups (LPS 1.0 mg/kg + OFE 0.3, 1.0 and 3.0 g/kg). After a 7‐d period of environmental adaptation, mice received an intraperitoneal injection of LPS (1.0 mg/kg) or saline 10 h before the behavioral tests. DLX, various doses of OFE (0.3, 1.0 and 3.0 g/kg), or distilled water were administered orally 1 h prior to the sucrose preference test (SPT).

SPT: Before the experiment, mice were housed individually in a single cage and trained to consume sucrose water for 2 days. On the initial day, each mouse had access to two bottles of aqueous solution containing 1% sucrose, with bottles was replaced every 12 h. On the second day, one bottle of water and one bottle of aqueous solution containing 1% sucrose were provided for an additional 24 h. The positions of the bottles were alternated every 12 h. After drug administration, each mouse was placed back into an individual cage and allowed to freely choose between two bottles, one containing an aqueous solution with 1% sucrose and the other containing water. The intake of sucrose and water in 12 h were recorded separately, and the sucrose preference can be calculated according to the following formular: sucrose preference (%) = [sugar consumption/ (sugar consumption + water consumption)] × 100%.

### 2.9 Monoamine assays by high‐performance liquid chromatography‐electrochemical detection (HPLC‐ECD)

The levels of 5‐HT and its metabolite 5‐hydroxyindole acetic acid (5‐HIAA) in the hippocampus of reserpine‐treated mice were measured using HPLC‐ECD method, as previously described (Xue et al., 2016). Briefly, tissue samples were homogenized in 0.4 M perchloric acid containing 2.0 mM Na_2_‐EDTA, 20.0 mM sodium citrate and 300.0 mM K_2_HPO_4_, and were centrifuged for 20 min at speed of 12,000 rpm at 4°C. The supernatant was collected and analyzed. The HPLC system, the mobile phase composition, and other specific details followed the protocol established previously (Xue et al., 2016). The content of 5‐HT and 5‐HIAA in the samples was quantified using a standard curve, with the area under the curve (AUC) serving as the index.

### 2.10 Western blotting analysis

Western blotting was used to measure the protein expression of 5‐HT_1A_, NF‐κB, and Iba‐1 in the hippocampus of reserpine‐treated mice and in LPS‐stimulated BV‐2 cells. Briefly, hippocampus tissue protein lysates were prepared using radioimmunoprecipitation assay (RIPA) lysis buffer (Servicebio Biotechnology, Wuhan, China) in the presence of protease inhibitors and phosphatase inhibitor. Protein samples (5 μL per well) were loaded onto 10% SDS‐polyacrylamide gels to separate the target proteins and transferred to polyvinylidene fluoride membranes (Millipore, Bedford, MA, USA). Subsequently, the membranes were washed with tris‐buffered saline containing 0.1% Tween‐20 (TBST), blocked with 5% non‐fat milk in TBST for 1 h at 37°C, and incubated with primary antibodies at 4°C overnight. After being washed three times with TBST, the membranes were incubated with the corresponding horseradish peroxidase‐conjugated secondary antibodies for 1 h at room temperature. Protein bands were visualized using an electrochemiluminescence system. Specifically, the bands were visualized using an Alpha Innotech alpha Ease FC luminescent image analyzer (AlphaInnotech, Santa Clara, CA, USA). The gray values of the protein bands were quantified using ImageJ software and normalized to the internal reference (*β*‐actin).

### 2.11 Elisa

The levels of TNF‐*α*, IL‐1*β*, and IL‐6 in hippocampal of reserpine‐treated mice and in LPS‐stimulated BV2 cell supernatant were measured using ELISA kits according to the attached instructions.

### 2.12 Determination of NO contents by Griess method

The level of NO in the cell culture supernatant was evaluated indirectly by measuring the contents of nitrous acid (NO_2_^−^) via the Griess reaction. After 24 h of treatment with LPS, the cell culture supernatant was collected. Then, 50 μL of the culture supernatant was mixed with 50 μL of Griess reagent I and II (Beyotime Biotech. Inc., Shanghai, China). The absorbance at 540 nm was measured using a microplate reader. The concentration of NO was determined according to the sodium nitrate standard curve.

### 2.13 Statistical analysis

Unless otherwise specified, statistical analysis was performed using GraphPad Prism (GraphPad Prism 9.0, Graph‐Pad Software Inc., San Diego, CA, USA), and the data are expressed as the mean ± SEM. The statistical significance of differences between groups was assessed using one‐way analysis of variance (ANOVA) followed by Dunnett's test. Student's *t*‐test was used to evaluate differences between two groups. Statistical significance was set at *p* < .05.

## 3 RESULTS

### 3.1 Chemical characteristics of OFE

**FIGURE 1:**
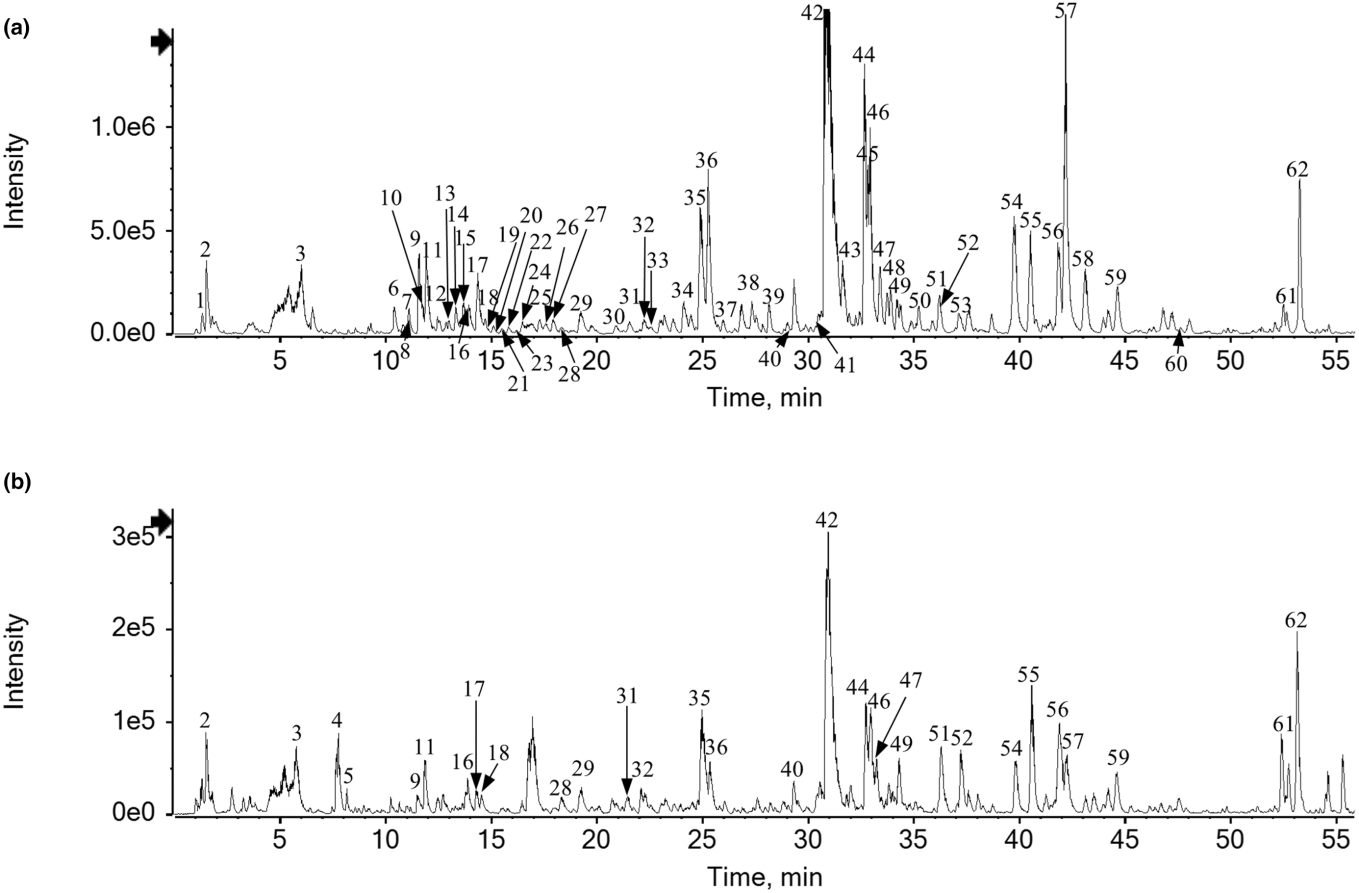
Chemical composition analysis and identification of OFE using UPLC‐Q‐TOF‐HRMS. (a) BPI chromatogram in negative ion mode of OFE. (b) BPI chromatogram in positive ion mode of OFE. UPLC‐Q‐TOF‐HRMS, Ultra‐performance liquid chromatography quadrupole time‐of‐flight high‐resolution mass spectrometry; BPI, base peak ion.

Ultra‐performance liquid chromatography quadrupole time‐of‐flight high‐resolution mass spectrometry (UPLC‐Q‐TOF‐HRMS) analysis was performed to identify the primary compounds of OFE. The peaks in the base peak ion (BPI) chromatogram were identified by comparing the recorded HRMS data to the calculated values and interpreting their tandem mass spectrometry (MS^2^) fragmentation patterns. This analysis was supported by an in‐house database and MS data reported in the literature (Kao et al., 2020; Liao et al., 2017). The BPI chromatograms of OFE in both negative and positive ionization modes are shown in Figure 1, and a total of 62 components were identified from OFE. One previous study characterized 36 compounds from *O. fragrans* roots using HPLC‐MS/MS (Liao et al., 2021), and another study reported 21 compounds identified from *O. fragrans* flowers using UPLC–QTOF–MS data (Zhou, Peng et al., 2017). Retention time (t_*R*_), adduct ions, molecular formulas, detected *m/z* values, error (*Δ*ppm), and MS^2^ data are listed in Table 1.

**TABLE 1:** Identified compounds from OFE by UPLC‐Q‐TOF‐HRMS.

No.	Tentative assignment	t_*R*_ (min)	Adduction	Formula	Detected (*m/z*)	Error (ppm)	MS^2^ data (*m/z*)
1	Allitol	1.30	[M − H]^−^	C_6_H_14_O_6_	181.0720	1.3	181.0707, 163.0623, 119.0346, 101.0242, 89.0243, 71.0138, 59.0139
2	Quinic acid	1.52	[M − H]^−^	C_7_H_12_O_6_	191.0566	2.6	191.0574, 131.0353, 103.0036, 85.0293
3	3‐{[6‐*O*‐(D‐galactopyranosyl)‐*β*‐D‐galactopyranosyl]oxy}‐1,2‐propanediyl diacetate	6.01	[M − H]^−^	C_19_H_32_O_15_	499.1667	−0.3	499.1667, 191.0559, 173.0455, 93.0343
4	Adenosine	7.72	[M + H]^+^	C_10_H_13_N_5_O_4_	268.1045	1.8	268.1053, 136.0620, 119.0353
5	Guanosine	8.15	[M + H]^+^	C_10_H_13_N_5_O_5_	284.0989	−0.2	153.0590, 152.0568, 135.0301, 110.0347
6	Vanilloloside	10.41	[M − H]^−^	C_14_H_20_O_8_	315.1080	−1.7	315.1081, 179.0556, 153.0552, 135.0452, 113.0247, 59.0140
7	Verbasoside	11.10	[M − H]^−^	C_20_H_30_O_12_	461.1672	1.6	461.1661, 315.1077, 297.0965, 135.0453, 113.0254
8	Neochlorogenic acid	11.19	[M − H]^−^	C_16_H_18_O_9_	353.0874	−1.1	353.0860, 191.0556, 179.0342, 135.0449, 85.0294
9	Salidroside	11.58	[M − H]^−^	C_14_H_20_O_7_	299.1134	−0.8	179.0584, 137.0609, 119.0512, 89.0255, 59.0144
10	Caffeic acid 4‐*O*‐*β*‐D‐glucopyranoside	11.74	[M − H]^−^	C_15_H_18_O_9_	341.0878	1.2	341.0875, 179.0355, 161.0252, 133.0302
11	Cistanoside F	11.94	[M − H]^−^	C_21_H_28_O_13_	487.1464	1.4	487.1446, 179.0351, 161.0246, 135.0453
12	Forsythoside E	12.46	[M − H]^−^	C_20_H_30_O_12_	461.1664	−0.1	461.1697, 315.1073, 205.0715, 163.0619, 143.0348, 135.0454
13	3‐*p*‐Coumaroylquinic acid	13.04	[M − H]^−^	C_16_H_18_O_8_	337.0926	−0.9	191.0568, 173.0458, 163.0405, 119.0505
14	Rhodioloside D	13.33	[M + HCOO]^−^	C_16_H_30_O_8_	395.1936	3.4	395.1918, 349.1859, 187.1341, 161.0449, 101.0239, 89.0242
15	Secoxyloganic acid	13.69	[M − H]^−^	C_16_H_22_O_11_	389.1086	−0.9	389.1083, 345.1183, 209.0449, 183.0663, 165.0559, 121.0663
16	Chlorogenic acid	13.95	[M − H]^−^	C_16_H_18_O_9_	353.0879	0.3	315.1075, 191.0562, 135.0454, 127.0402
17	6‐[(2*E*)‐3‐(4‐hydroxyphenyl)‐2‐propenoate]‐*β*‐D‐Glucopyranose	14.36	[M − H]^−^	C_15_H_18_O_8_	325.0926	−0.9	325.0918, 265.0697, 145.0292, 117.0343, 59.0137
18	(1*R*,2*R*,4*S*)‐*p*‐menthane‐1,2,8‐triol 8‐glucoside	14.71	[M + HCOO]^−^	C_16_H_30_O_8_	395.1915	−2.0	395.1927, 349.1867, 187.1340, 179.0565, 89.0251, 59.0143
19	Cryptochlorogenic acid	14.84	[M − H]^−^	C_16_H_18_O_9_	353.0875	−0.9	353.0874, 191.0560, 179.0348, 173.0455, 135.0451
20	Esculetin	15.21	[M − H]^−^	C_9_H_6_O_4_	177.0195	0.9	177.0185, 149.0265, 133.0294, 105.0347, 89.0389
21	3‐Methyl‐4‐*O*‐(6′‐*O*‐*α*‐L‐rhamnosyl)‐*β*‐D‐glucopyranosyloctanoic acid	15.50	[M + HCOO]^−^	C_21_H_38_O_12_	527.2357	2.2	527.2365, 481.2302, 349.1861, 221.0662, 191.0561
22	Caffeic acid	15.82	[M − H]^−^	C_9_H_8_O_4_	179.0356	3.4	179.0344, 135.0448, 117.0346, 79.0545
23	(2*S*)‐2,6,7‐Trihydroxy‐7‐methyl‐3‐methyleneoctyl *β*‐D‐glucopyranoside	16.20	[M − H]^−^	C_16_H_30_O_9_	365.1827	2.7	365.1789, 203.1277, 119.0343, 89.0239, 59.0136
24	Ferulic acid 4‐*O*‐*β*‐glucopyranoside	16.48	[M − H]^−^	C_16_H_20_O_9_	355.1027	−2.1	355.1017, 175.0401, 160.0168, 132.0220, 59.0141
25	Methyl glucooleoside	17.29	[M + HCOO]^−^	C_23_H_34_O_16_	611.1829	0	565.1786, 403.1246, 371.0971, 223.0608, 179.0559, 165.0552
26	Roseoside	17.57	[M + HCOO]^−^	C_19_H_30_O_8_	431.1920	−0.6	431.1907, 385.1855, 223.1337, 205.1235, 153.0922
27	Tuberonic acid glucoside	17.94	[M − H]^−^	C_18_H_28_O_9_	387.1655	−1.4	387.1660, 207.1022, 163.1125, 89.0241, 59.0137
28	Coniferin	18.32	[M − H]^−^	C_16_H_22_O_8_	341.1236	−1.7	299.1146, 281.1019, 119.0501, 101.0244, 59.00139
29	5‐*O*‐*p*‐Coumaroylquinic acid	19.25	[M − H]^−^	C_16_H_18_O_8_	337.0936	2.1	191.0563, 173.0453, 165.0559, 163.0400, 93.0341
30	3,4‐Diacetyloxy‐L‐phenylalanine	20.90	[M − H]^−^	C_13_H_15_NO_6_	280.0825	−0.6	280.0829, 132.0310, 88.0408
31	Isopropyl 3‐(3,4‐dihydroxyphenyl)‐2‐hydroxypropanoate	21.53	[M − H]^−^	C_12_H_16_O_5_	239.0928	1.3	239.0924, 195.1013, 141.0568, 97.0657, 59.0139
32	5‐Feruloylquinic acid	22.24	[M − H]^−^	C_17_H_20_O_9_	367.1047	3.4	367.1009, 191.0553, 173.0444, 134.0368
33	*p*‐Coumaric acid	22.54	[M − H]^−^	C_9_H_8_O_3_	163.0406	3.3	163.0415, 119.0503, 93.0353
34	Betulalbuside A	24.12	[M + HCOO]^−^	C_16_H_28_O_7_	377.1811	−1.6	377.1798, 331.1730, 179.0563, 89.0243, 59.0135
35	*S*‐Suspensaside	24.89	[M − H]^−^	C_29_H_36_O_16_	639.1938	1.2	639.1916, 621.1813, 179.0345, 161.0241, 151.0391
36	*R*‐Suspensaside	25.27	[M − H]^−^	C_29_H_36_O_16_	639.1924	−1.0	639.1888, 621.1782, 179.0336, 161.0238, 151.0392
37	Ferulic acid	25.80	[M − H]^−^	C_10_H_10_O_4_	193.0507	0.3	193.0539, 178.0295, 149.0622, 134.0376, 133.0298
38	Zingiberoside B	27.34	[M + HCOO]^−^	C_21_H_36_O_11_	509.2249	1.8	509.2245, 463.2185, 331.1760, 179.0561
39	*β*‐Oxoacteoside	28.15	[M − H]^−^	C_29_H_34_O_16_	637.1763	−1.7	637.1776, 475.1467, 329.0882, 161.0250
40	Rutin	29.28	[M − H]^−^	C_27_H_30_O_16_	609.1469	1.3	609.1475, 447.1487, 301.0320, 300.0257, 271.0224
41	Luteoloside	30.32	[M − H]^−^	C_21_H_20_O_11_	447.0945	2.7	285.0391, 269.1348, 209.1185, 59.0135
42	Verbascoside	30.88	[M − H]^−^	C_29_H_36_O_15_	623.1988	1.1	623.1997, 461.1675, 161.0249, 135.0456
43	Sphaerophyside SC	31.69	[M − H]^−^	C_20_H_30_O_10_	429.1772	1.3	429.1738, 267.1225, 249.1120, 223.1333, 161.0972
44	Ilicifolioside A	32.67	[M − H]^−^	C_31_H_40_O_16_	667.2261	2.6	667.2238, 621.1812, 459.1503, 179.0344, 161.0242, 151.0396
45	Isoacteoside	32.82	[M − H]^−^	C_29_H_36_O_15_	623.1991	1.5	623.1975, 461.167, 161.0253, 133.0293
46	2‐(3,4‐Dihydroxyphenyl)‐2‐ethoxyethyl 3‐*O*‐(6‐deoxy‐*α*‐L‐mannopyranosyl)‐*β*‐D‐glucopyranoside 4‐[(2*E*)‐3‐(3,4‐dihydroxyphenyl)‐2‐propenoate]	32.93	[M − H]^−^	C_31_H_40_O_16_	667.2255	1.7	667.2247, 621.1828, 459.1516, 179.0349, 161.0250
47	Forsythoside I	33.40	[M − H]^−^	C_29_H_36_O_15_	623.1991	1.5	623.1983, 461.1660, 315.1082, 161.0248, 135.0456
48	Syringalide A 3′‐*α*‐L‐rhamnopyranoside	33.90	[M − H]^−^	C_29_H_36_O_14_	607.2054	3.6	607.2034, 445.1705, 161.0241, 133.0289
49	Byzantionoside B	34.20	[M + HCOO]^−^	C_19_H_32_O_7_	417.2139	2.1	417.2100, 373.1852, 209.1185, 59.0137
50	(2*S*)‐2‐(3,4‐dihydroxyphenyl)‐2‐ethoxyethyl 6‐*O*‐(6‐deoxy‐α‐L‐mannopyranosyl)‐,4‐[(2*Z*)‐3‐(3,4‐dihydroxyphenyl)‐2‐propenoate]‐*β*‐D‐glucopyranoside	35.23	[M − H]^−^	C_31_H_40_O_16_	667.2269	1.7	667.2271, 621.1836, 487.1465, 459.1512, 251.0559, 179.0348, 161.0245
51	Cissoic acid	36.21	[M − H]^−^	C_20_H_30_O_10_	429.1772	1.3	429.1747, 385.1842, 249.1121, 205.1226, 161.1331
52	2‐Acetylacteoside	36.25	[M − H]^−^	C_31_H_38_O_16_	665.2097	1.5	665.2111, 623.1964, 461.1657, 161.0246
53	Oleuropein	37.12	[M − H]^−^	C_25_H_32_O_13_	539.1789	3.5	539.1736, 377.1203, 307.0781, 275.0922, 254.0914, 89.0240
54	Oleoacteoside	39.75	[M − H]^−^	C_46_H_58_O_25_	1009.3221	−4.5	1009.3167, 847.2276, 665.2070, 623.1965, 461.1649, 161.0245
55	Phillyrin	40.52	[M + HCOO]^−^	C_27_H_34_O_11_	579.2099	2.7	579.2071, 371.1494, 356.1239, 161.0449
56	10‐Acetoxyligustroside	41.81	[M − H]^−^	C_27_H_34_O_14_	581.1885	1.6	581.2307, 537.1704, 419.1369, 291.0879, 179.0366
57	Ligustroside	42.18	[M − H]^−^	C_25_H_32_O_12_	523.1830	1.7	523.1813, 361.1285, 291.0873, 259.0972, 127.0399, 101.0238
58	4‐[(2*R*,3*R*,4*S*,5*S*,6*R*)‐3,4,5‐trihydroxy‐6‐(hydroxymethyl)oxan‐2‐yl]oxypentan‐2‐yl (*E*)‐3‐(4‐hydroxyphenyl)prop‐2‐enoate	43.11	[M − H]^−^	C_20_H_28_O_9_	411.1670	2.3	411.1680, 249.1146, 205.1241, 187.1134, 161.1336, 89.0242
59	Geranyl *β*‐primeveroside	44.64	[M + HCOO]^−^	C_21_H_36_O_10_	493.2310	4.0	493.2247, 447.2234, 315.1815, 179.0562, 149.0449, 89.0242
60	Naringenin	47.98	[M − H]^−^	C_15_H_12_O_5_	271.0614	0.7	271.0587, 177.0189, 151.0032, 119.0494, 107.0136, 65.0024
61	Floraosmanoside II	52.62	[M + HCOO]^−^	C_24_H_42_O_10_	535.2762	0.4	535.2767, 489.2718, 357.2277, 161.0450
62	Floraosmanoside I	53.26	[M + HCOO]^−^	C_24_H_42_O_10_	535.2755	−0.9	535.2823, 489.2734, 357.2281, 161.0544, 101.0245

Abbreviations: MS, mass spectra; *m/z*, mass‐to‐charge ratio; ppm, part per million; t_*R*_, retention time; UPLC‐Q‐TOF‐HRMS, Ultra‐performance liquid chromatography quadrupole time‐of‐flight high‐resolution mass spectrometry.

The identified compounds can be classified as lignans, terpenoids, flavonoids, organic acids, secoiridoids, alkaloids, and other compounds. Glycoside compounds were shown to be the major constituents of OFE. Phenylethanoid glycoside derivatives (compounds **7**, **9**, **12**, **42**, **45**, **47**, and **52**), secoiridoid derivatives (compounds **15**, **25**, **53**, **54**, **56**, and **57**), terpene glycoside derivatives (compounds **21**, **23**, **26**, **27**, **34**, **38**, **59**, **61**, and **62**), and phenolic glycosides (compounds **6**, **11**, **17**, **24**, **35**, **36**, **43**, and **48**) were characterized and identified from OFE.

### 3.2 Effects of OFE on the TST, FST, and LAT in mice

**FIGURE 2:**
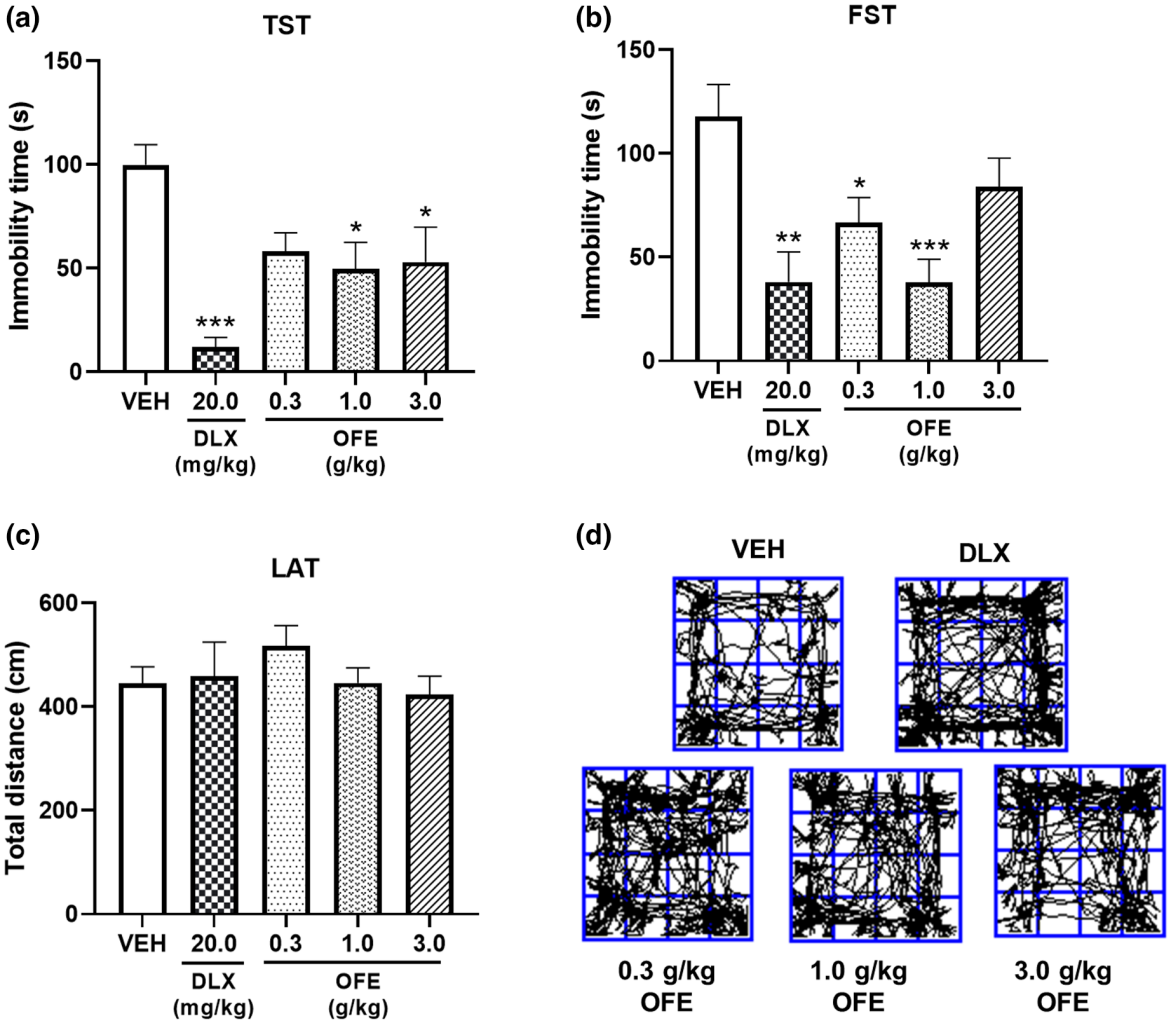
Effects of acute treatment with OFE on behavioral despair tests in mice. (a, b) Immobility time in the FST and TST. (c, d) Total distance and representative tracks in the LAT. OFE (0.3, 1.0, and 3.0 g/kg), DLX (20.0 mg/kg), and equivolume vehicle were administrated orally to mice 60 min before testing. Mice were subjected to FST, TST, and LAT. Data are presented as the mean ± SEM (*n* = 6–10). *p < .05, **p < .01, ***p < .001 vs. vehicle. FST, forced swim test; TST, tail suspension test; LAT, locomotor activity test.

The FST and TST were initially conducted to rapidly evaluate the antidepressant‐like effects of acute pretreatment with OFE in mice. Figure 2a shows the effects of OFE on the immobility time in the TST on mice. Compared with the vehicle, the oral administration of DLX at 20.0 mg/kg significantly decreased the immobility time (*p* < .001), indicating that the behavioral tests were performed under normal conditions and the results were reliable. Oral administration of OFE (1.0 and 3.0 g/kg) significantly reduced the immobility time (*p* < .05). Figure 2b shows the effects of OFE on the immobility time in the FST on mice. Compared with the vehicle, the acute administration of DLX (20.0 mg/kg) significantly reduced the immobility time (*p* < .01). In addition, acute oral treatment with OFE (0.3 and 1.0 g/kg) significantly decreased the immobility time (*p* < .05 for 0.3 g/kg, *p* < .001 for 1.0 g/kg).

Subsequently, the results of the LAT were analyzed to eliminate any false‐positive outcomes that can potentially be attributed to a central stimulation effect of OFE. The acute oral administration of OFE at various doses (*p* > .05) and DLX (*p* > .05) had no effects on the locomotor activity of mice (Figure 2c,d).

### 3.3 Effects of OFE on 5‐HTP induced head‐twitch behavior

**FIGURE 3:**
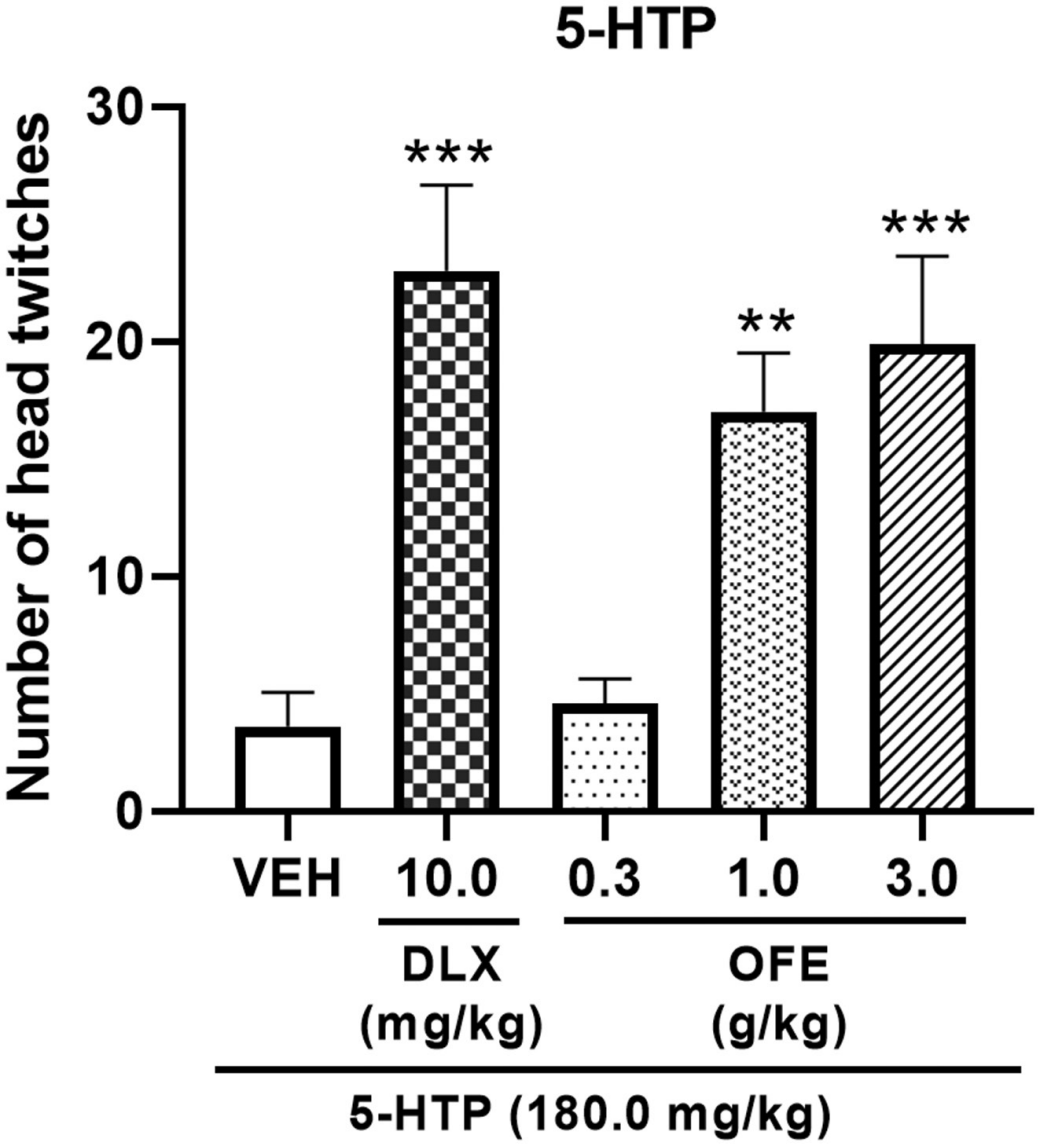
Effects of OFE on 5‐HTP‐induced head‐twitch behavior in mice. OFE (0.3, 1.0, and 3.0 g/kg), DLX (20.0 mg/kg), and equivolume vehicle were administrated orally to mice 60 min before 5‐HTP (180.0 mg/kg) injection. Data are presented as the mean ± SEM (n = 10). **p < .01, ***p < .001 vs. vehicle. 5‐HTP, 5‐hydroxytryptophan.

As shown in Figure 3, compared with the vehicle group, OFE (1.0 and 3.0 g/kg) significantly increased the number of head twitches in mice (*p* < .01 for 1.0 g/kg, *p* < .001 for 3.0 g/kg). DLX at 10.0 mg/kg also significantly increased the number of 5‐HTP‐induced head twitches in mice (*p* < .001).

### 3.4 Effects of OFE on yohimbine induced lethal toxicity

As shown in Table 2, yohimbine injection only induced 10% mortality rate in naïve mice, whereas the combined administration of DLX (20.0 mg/kg) with yohimbine significantly increased the mortality rate to 90% (*p* < .01). Pretreatment with OFE at various doses did not have significant effects on yohimbine induced toxicity (*p* > .05).

**TABLE 2:** Effects of OFE on mortality rate in the yohimbine toxicity potentiation test on mice.

Group	Dose/(g/kg)	Death numbers after 24 h	Mortality rate after 24 h/(%)
VEH	–	1	10
DLX	0.02	9	90**
OFE	0.30	0	0
	1.00	3	30
	3.00	2	20

Abbreviations: DLX, duloxetine; VEH, vehicle.

** *p* < .01 vs. vehicle, Fisher's exact test. *n* = 10.

### 3.5 Effects of OFE on the function of the monoamine system in reserpine‐treated mice

#### 3.5.1 Effects of OFE on reserpine induced ptosis, akinesia, and hypothermia

As shown in Table 3, administration of reserpine at a dose of 5.0 mg/kg effectively induced ptosis, akinesia, and hypothermia in mice (*p* < .001). Pretreatment with OFE at a dose of 3.0 g/kg significantly ameliorated reserpine‐induced ptosis in mice after 1 h of reserpine injection (*p* <.001). However, OFE at doses of 1.0 and 3.0 g/kg did not cause significant improvement in akinesia and hypothermia in reserpine‐treated mice (*p* >.05). In contrast, DLX at a dose of 20.0 mg/kg significantly ameliorated ptosis and hypothermia in reserpine‐treated mice (*p* < .001 for blepharoptosis, *p* < .01 for hypothermia).

**TABLE 3:** Effects of acute treatment with OFE on reserpine‐induced ptosis, hypothermia, and akinesia in mice.

Group	Dose (g/kg)	Ptosis score after 1 h	Percentage with akinesia (%)	Rectal temperature (°C)
VEH	–	0	0	0.62 ± 0.18
MOD	–	2.80 ± 0.33***	90***	2.30 ± 0.31***
DLX	0.02	0^###^	100	1.25 ± 0.12^##^
OFE	1.00	2.10 ± 0.18	60	2.05 ± 0.29
	3.00	1.40 ± 0.31^###^	70	1.70 ± 0.11

*Note*: Data are presented as the mean ± SEM (*n* = 10).

Abbreviations: DLX, duloxetine; MOD, model; VEH, vehicle;

*** *p* < .001 vs. vehicle;

^##^ *p* < .01.

^###^ *p* < .001 vs. model.

#### 3.5.2 Effects of OFE on reserpine‐induced 5‐HT and 5‐HIAA changes

**FIGURE 4:**
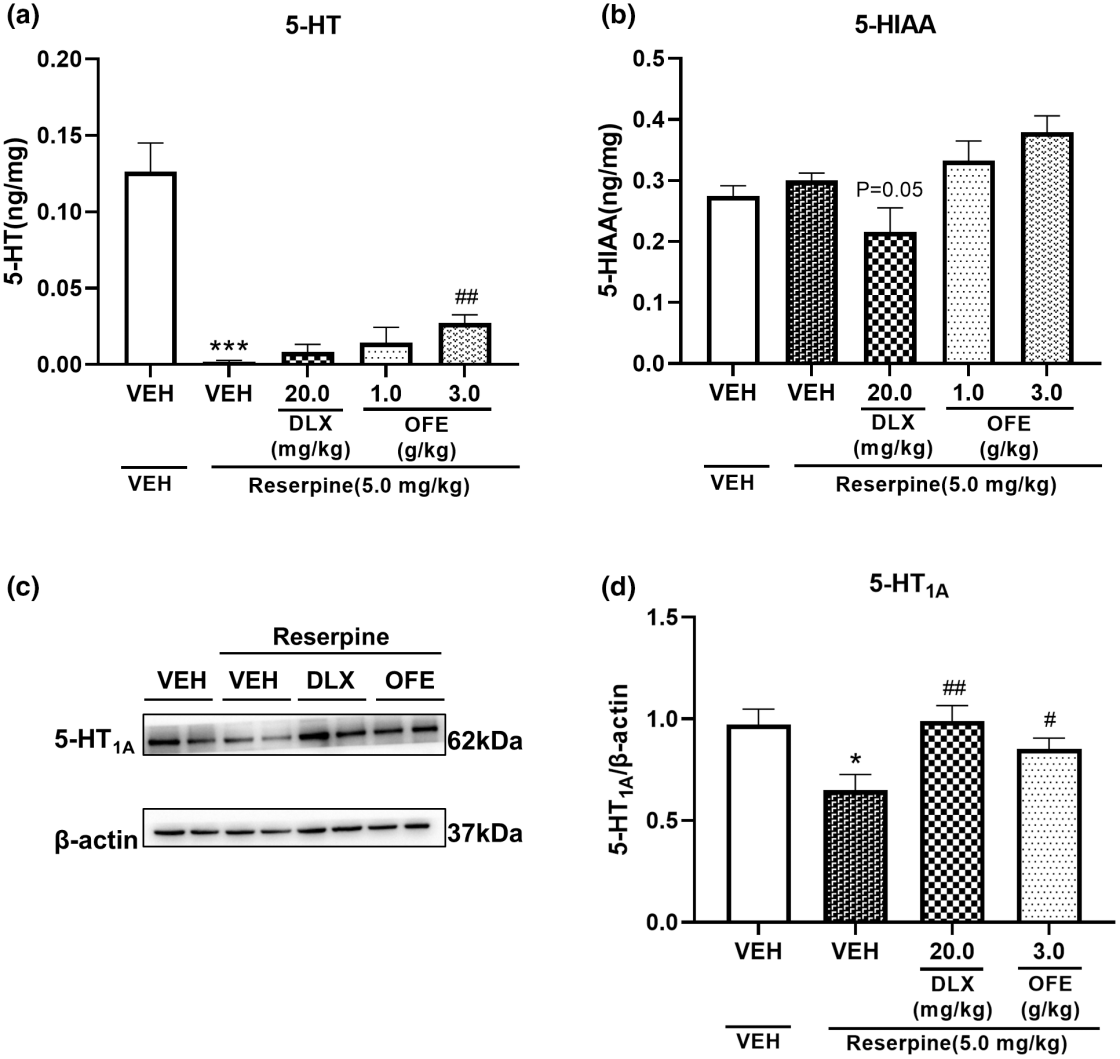
Effects of OFE on 5‐HT (a) and 5‐HIAA (b) levels, 5‐HT_1A_ protein expression (c); quantitative analysis of 5‐HT_1A_ expression levels (d) in the hippocampus of reserpine‐treated mice. Data are presented as the mean ± SEM (*n* = 8–10). *p < .05, ***p < .001 vs. vehicle; ^#^ *p* < .05, ^##^ *p* < .01vs. model. 5‐HT, 5‐hydroxytryptamine; 5‐HIAA, 5‐hydroxyindole acetic acid.

Figure 4a,b illustrates the effects of OFE and DLX on the levels of 5‐HT and its metabolite 5‐HIAA in the hippocampus of reserpine‐treated mice. Compared with the vehicle, injection of reserpine at 5.0 mg/kg significantly decreased the 5‐HT levels and increased the 5‐HIAA levels in the hippocampus (*p* <.001 for 5‐HT; *p* >.05 for 5‐HIAA). OFE at a dose of 3.0 g/kg significantly reversed the decline in 5‐HT levels induced by reserpine (*p* <.01). However, OFE at doses of 1.0 and 3.0 g/kg had no effect on 5‐HIAA levels (*p* >.05). DLX at a dose of 20.0 mg/kg significantly reversed the increase in 5‐HIAA levels induced by reserpine (*p* =.05).

#### 3.5.3 Effects of OFE on 5‐HT1A levels in the hippocampus of reserpine‐treated mice

We investigated the effects of OFE on 5‐HT_1A_ expression in reserpine‐treated mice using western blotting. As shown in Figure 4c,d, compared with the vehicle, injection of reserpine at a dose of 5.0 mg/kg significantly downregulated 5‐HT_1A_ expression (*p* < .05), whereas pretreatment with DLX at a dose of 20.0 mg/kg or OFE at a dose of 3.0 g/kg successfully restored the expression of 5‐HT_1A_ to normal levels (*p* < .01 for DLX; *p* < .05 for OFE 3.0 g/kg).

### 3.6 Effects of OFE on proinflammatory cytokine levels in reserpine‐treated mice

**FIGURE 5:**
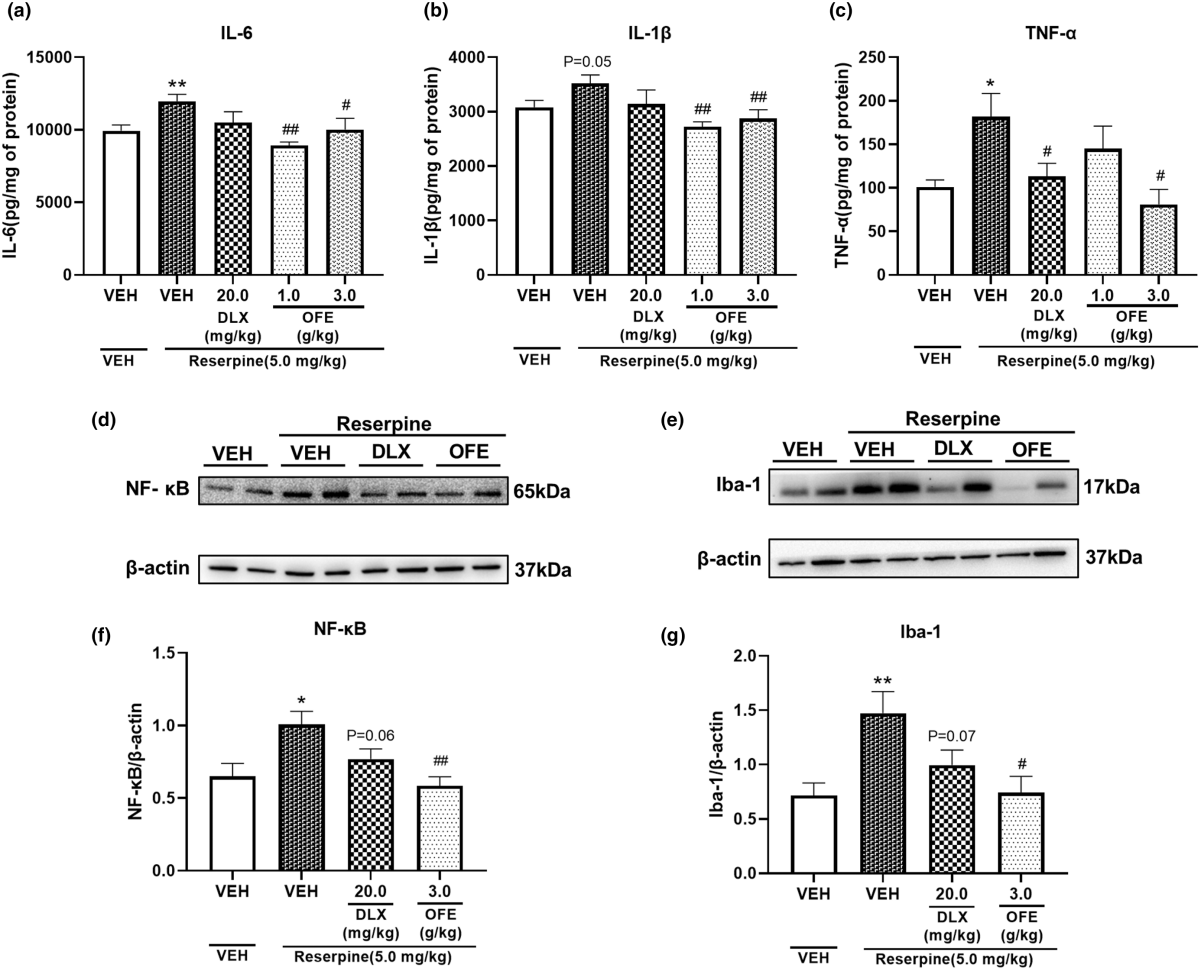
Effects of OFE on the inflammatory response in the hippocampus of reserpine‐treated mice. (a–c) IL‐6, IL‐1β, and TNF‐α levels. Data are presented as the mean ± SEM (*n* = 6–10). *p < .05, **p < .01, vs. vehicle; ^#^ *p* < .05, ^##^ *p* < .01 vs. model. NF‐κB and Iba‐1 protein expression (d, e); quantitative analysis of NF‐κB and Iba‐1 expression levels (f, g) in the hippocampus of reserpine‐treated mice. Data are presented as the mean ± SEM (*n* = 6). *p < .05, **p < .01, vs. vehicle; ^#^ *p* < .05, ^##^ *p* < .01 vs. model. IL‐6, interleukin‐6; IL‐1β, interleukin‐1β; TNF‐α, tumor necrosis factor‐α; NF‐κB, nuclear factor kappa beta.

Figure 5a–c illustrate the effects of OFE and DLX on the proinflammatory cytokine levels, including IL‐6, IL‐1*β*, and TNF‐*α*, in the hippocampus of reserpine‐treated mice. Compared with the vehicle, pretreatment with reserpine at 5.0 mg/kg significantly increased the IL‐6, IL‐1*β*, and TNF‐*α* levels in the hippocampus (*p* < .01 for IL‐6; *p* = .05 for IL‐1*β*; *p* < .05 for TNF‐*α*). Treatment with OFE at doses of 1.0 or 3.0 g/kg significantly decreased the IL‐6 (*p* < .01 for 1.0 g/kg, *p* < .05 for 3.0 g/kg), IL‐1*β* (*p* < .01 for 1.0 and 3.0 g/kg), and TNF‐*α* (*p* < .05 for 3.0 g/kg) levels. In addition, treatment with DLX at a dose of 20.0 mg/kg significantly decreased the TNF‐*α* level (*p* < .05).

### 3.7 Effects of OFE on NF‐κB and Iba‐1 levels in the hippocampus of reserpine‐treated mice

Figure 5d–g illustrate the effects of OFE on NF‐κB and Iba‐1 levels in the hippocampus of reserpine‐treated mice. Compared with the vehicle, pretreatment of reserpine at a dose of 5.0 mg/kg significantly upregulated NF‐κB expression (*p* < .05) and Iba‐1 (*p* < .01), whereas treatment with OFE at a dose of 3.0 g/kg successfully restored NF‐κB expression to normal levels (*p* < .01) and Iba‐1 (*p* < .05).

### 3.8 Effects of OFE on NO and proinflammatory cytokine secretion in LPS‐stimulated BV‐2 microglial cells

**FIGURE 6:**
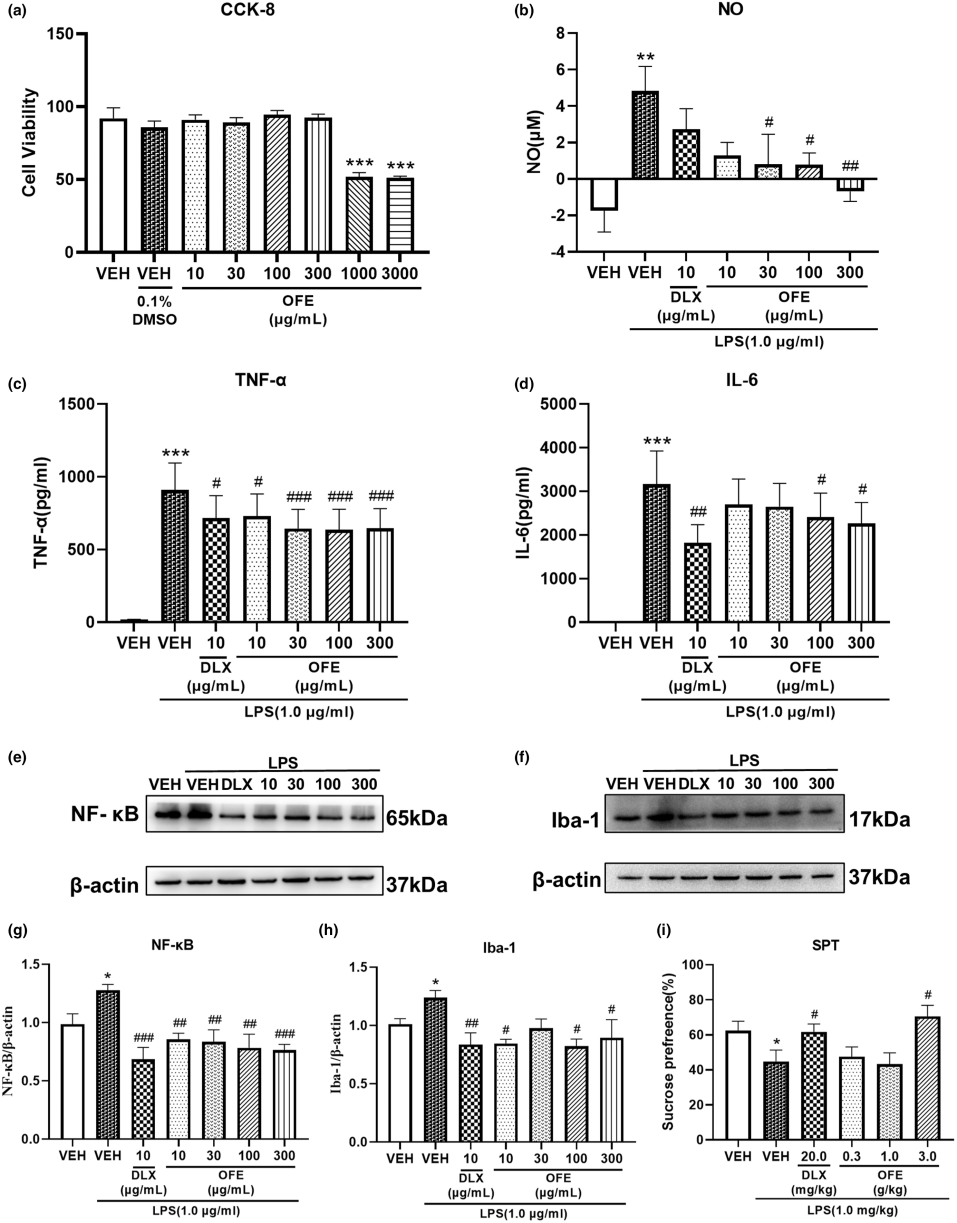
Effects of OFE on LPS‐induced depression‐like mice and on the inflammatory response in microglial cells stimulated with LPS. (a) Effects of OFE on BV‐2 microglial cell viability. BV‐2 cells were treated with 10–3000 μg/mL OFE for 24 h, then cell viability was evaluated using the CCK‐8 assay. Data are presented as the mean ± SEM (n = 6). ***p < .001 vs. vehicle. (b) Effects of OFE on NO production in culture supernatant of BV2 microglial cells stimulated with LPS. Data are presented as the mean ± SEM (*n* = 6). **p < .01 vs. vehicle; ^#^ *p* < .05, ^##^ *p* < .01, vs. model. (c, d) Effects of OFE on TNF‐α (c) and IL‐6 (d) levels in BV‐2 microglial cells stimulated with LPS. TNF‐α and IL‐6 levels in cell culture supernatants were evaluated using ELISA kits. Data are presented as the mean ± SEM (n = 6). ***p < .001 vs. vehicle; ^#^ *p* < .05, ^##^ *p* < .01, ^###^ *p* < .001, vs. model. NF‐κB and Iba‐1 protein expression (e, f); quantitative analysis of NF‐κB and Iba‐1 expression levels (g, h). NF‐κB and Iba‐1 expression levels are shown as a ratio relative to β‐Actin. Data are presented as the mean ± SEM (*n* = 5). *p < .05 vs. vehicle; ^#^ *p* < .05, ^##^ *p* < .01, ^###^ *p* < .001, vs. model. (i) Performance in the SPT. Data are presented as the mean ± SEM (*n* = 14–15). *p < .05 vs. vehicle; ^#^ *p* < .05, vs. model. LPS, lipopolysaccharide; CCK‐8, cell counting kit‐8; TNF‐α, tumor necrosis factor‐α; IL‐6, interleukin‐6; ELISA, enzyme‐linked immunosorbent assay; NF‐κB, nuclear factor kappa beta; Iba‐1, ionized calcium binding adaptor molecule 1; SPT, sucrose preference test.

We evaluated the cytotoxicity of OFE in BV‐2 cells using CCK‐8 assay. As shown in Figure 6a, OFE concentrations of >300 μg/mL significantly inhibited cell proliferation. Therefore, we selected an OFE concentration ranging 10–300 μg/mL for subsequent experiments. Next, we investigated whether OFE had antagonistic effects on LPS‐stimulated inflammation in BV‐2 microglial cells. Figure 6b–d illustrate the effects of OFE on the LPS‐stimulated production of NO, TNF‐α, and IL‐6. Consistent with previous studies, LPS at a concentration of 1.0 μg/mL significantly increased NO, TNF‐α, and IL‐6 expression (*p* < .01, *p* < .001, *p* < .001). Pre‐treatment with OFE resulted in a significant reduction in the production of inflammatory mediators, including NO (*p* < .05 for 30 μg/mL, *p* < .05 for 100 μg/mL and *p* < .01 for 300 μg/mL), TNF‐α (*p* < .05 for 10 μg/mL and *p* < .001 for 30–300 μg/mL), and IL‐6 (*p* < .05 for 100 and 300 μg/mL), in a dose‐depended manner. DLX exhibited similar antagonistic effects as those of DLX on TNF‐α and IL‐6 expression (*p* < .05 for TNF‐α, *p* < .01 for IL‐6).

### 3.9 Effects of OFE on NF‐KB and IBA‐1 levels in LPS‐stimulated BV‐2 microglial cells

Figure 6e–h show the effects of OFE on NF‐κB and Iba‐1 expression in LPS‐ stimulated BV‐2 cells. Compared with the control group, the LPS‐stimulated group showed significantly upregulated NF‐κB (*p* < .05) and Iba‐1 (*p* < .05) expression. However, pretreatment with OFE significantly reversed NF‐κB (*p* < .01 for 10–100 μg/mL, *p* < .001 for 300 μg/mL) and Iba‐1 (*p* < .05 for 10, 100 and 300 μg/mL) expression levels in BV‐2 cells, restoring them to normal levels. DLX exhibited similar antagonistic effects on NF‐κB and Iba‐1 expression (*p* < .001 for NF‐κB, *p* < .01 for Iba‐1).

### 3.10 Effects of OFE on LPS‐induced depression‐like behavior

As illustrated in Figure 6i, acute exposure to LPS (1.0 mg/kg) led to a significant decrease in sucrose preference in mice compared with vehicle mice (*p* < .05). However, treatment with DLX (20.0 mg/kg) and OFE (3.0 g/kg) successfully restored the sucrose preference in mice to normal levels (*p* < .05 for DLX; *p* < .05 for OFE 3.0 g/kg).

## 4 DISCUSSION

The present study demonstrated that (1) A single dose of OFE exerts antidepressant‐like effects in both behavioral despair and LPS‐induced depressive mouse models. (2) The antidepressant‐like effects of OFE are mediated by an increase in 5‐HT levels and suppression of neuroinflammation. (3) UPLC‐Q‐TOF‐HRMS analysis showed that the OFE was primarily composed of glycoside derivatives, which may be important components that contribute to the antidepressant effects of OFE.

In the present study, we first used behavioral despair mouse models to investigate whether OFE had antidepressant‐like activity. Behavioral despair models, including the TST and FST, were convenient and predictive for antidepressant screening (Castagné et al., 2011). In 2015, Jawaid et al. published the first report related to the antidepressant effects of *O. fragrans* in behavioral despair models (Jawaid et al., 2015). Some differences exist between their study and ours. For instance, they investigated the antidepressant‐like effects of the fruits, whereas we investigated the antidepressant effects of the flowers of *O. fragrans*. In addition, they found that successive administration of the ethanolic extract of the fruits of *O. fragrans* for 7 days exhibited antidepressant‐like activity in the TST and FST in mice, whereas in our study, a single administration of OFE exhibited significant antidepressant effects in the TST and FST in mice. To the best of our knowledge, this is the first study to demonstrate that a single dose of OFE can exert antidepressant‐like effects in behavioral despair models. Because stimulants of the central nervous system (CNS), such as caffeine and amphetamine, can reduce the immobility time in the TST and FST, we evaluated the effects of OFE on locomotor activity through the LAT. We observed that OFE, administered at a dose within the pharmacologically effective range, did not affect locomotor activity, thereby excluding the false‐positive possibility induced by CNS stimulation.

Next, we studied the potential mechanisms underlying the antidepressant effects of OFE, focusing on the monoamine hypothesis. The monoamine hypothesis is a popular hypothesis explaining depression pathophysiology and guiding the discovery of antidepressant therapeutics. The monoamine hypothesis proposes that the occurrence of depression may be closely linked to an abnormal deficiency in monoaminergic neurotransmitters, including 5‐HT, norepinephrine (NE), and dopamine (DA) (Jiang et al., 2022; Schildkraut et al., 1967). We used two pharmacological models, that is, 5‐HTP‐induced head‐twitch and yohimbine toxicity potentiation tests, to investigate whether OFE can enhance the function of 5‐HT and the NE transmitter system. Notably, 5‐HTP is a precursor of 5‐HT, which can pass through the blood–brain barrier. Peripheral injection of 5‐HTP rapidly increases 5‐HT levels in the brain, and subsequently induces head‐twitch behavior through 5‐HT_2A_ receptor activation (Corne et al., 2012). Based on our results, OFE potentiated 5‐HTP‐induced head‐twitch behavior, suggesting that it may have enhancing effects on 5‐HTergic function. In contrast, OFE did not affect yohimbine induced mortality, indicating that NEergic enhancement may not be involved in its antidepressant activity. Yohimbine can enhance NE release in the synaptic cleft by antagonizing the α2 auto‐receptor; therefore, co‐administration of yohimbine with a NE enhancement drug should increase the mortality rate as a result of NE overdose (Malick, 1983).

To further support the elucidated effects of OFE on the monoaminergic transmitter system, we developed a reserpine‐induced monoamine depletion mouse model. Reserpine is an inhibitor of vesicular monoamine transporter 2 (VMAT2), and functions in pumping monoaminergic transmitters (5‐HT, NE, and DA) into pre‐synaptic storage vesicles. Therefore, reserpine treatment leads to the changing of 5‐HT, NE, and DA into their metabolites, and subsequently results in various behavior deficits and conditions such as ptosis, hypothermia, and akinesia (Antkiewicz‐Michaluk et al., 2014). In the present study, we observed that OFE only reversed ptosis behavior, which was induced by 5‐HT depletion following reserpine treatment, supporting that OFE activated CNS 5‐HTergic function in vivo. HPLC‐ECD assays further validated that single‐dose OFE administration selectively restored 5‐HT depletion in the hippocampus in reserpine‐treated mice, whereas NE and DA levels remained unaffected after OFE administration. Interestingly, our study indicated that OFE and DLX may exhibit various effects on the 5‐HT system. We observed that DLX, a dual 5‐HT and NE reuptake inhibitor, antagonized the reserpine‐induced increase in 5‐HIAA without affecting 5‐HT concentrations in the hippocampus, which was consistent with the findings of our previous study (Xue et al., 2016). Similarly, Fuller et al. reported that DLX exerted dose‐dependent lowering effects on 5‐HIAA in mouse brains without altering 5‐HT levels, which is considered a characteristic of a 5‐HT reuptake inhibitor (Fuller et al., 1994). In contrast, OFE primarily increased 5‐HT levels. Several traditional herbal medicines have been reported to regulate 5‐HTergic function by increasing 5‐HT contents instead of altering 5‐HT metabolism (Abbasi‐Maleki & Mousavi, 2017; Ghosh et al., 2021; Navidi & Abbasi, 2023). Park et al. reported that a representative herbal medicine, Bangpungtongsung‐San, significantly reversed the decrease in 5‐HT levels in the brain caused by the long‐term injection of reserpine (Park et al., 2020). Interestingly, ginsenoside Rb1, a major chemical component of ginseng, was reported to improve LPS‐induced depressive behavior in mice by increasing 5‐HT and tryptophan (TRP) expression in the hippocampus (Liang et al., 2022). Collectively, our study demonstrated for the first time that increasing 5‐HT levels in the brain may be involved in the antidepressant‐like effects of OFE. In addition to causing monoamine depletion, Park et al. showed that chronic administration of low‐dose reserpine (0.5 mg/kg) for 10 days induced neuroinflammation (Park et al., 2020). Our present study showed that a single injection of high‐dose reserpine (5 mg/kg) also resulted in an inflammatory response in the brain, as evidenced by the activation of microglial cells and increased production of pro‐inflammatory cytokines (IL‐6, TNF‐α, and IL‐1β). Interestingly, 5‐HT exerts immunoregulatory activity by modulating serotonin receptors, with the 5‐HT_1A_ receptor playing a critical role in this process (Wu et al., 2019). A recent study by Vašíček et al. showed that 5‐HT inhibited IL‐6, TNF‐α, and NO production in murine macrophages of the RAW264.7 cell line when stimulated with 100 ng/mL LPS. Moreover, Freire‐Garabal et al. reported that serotonin can enhance the activity of peritoneal macrophages by upregulating 5‐HT_1A_ receptor expression (Freire‐Garabal et al., 2009). Our present study determined that 5‐HT_1A_ expression was downregulated in response to reserpine‐induced 5‐HT depletion, which was reversed after administration of OFE. Therefore, we aimed to determine whether OFE can exert anti‐inflammatory effects by regulating 5‐HT and 5‐HT_1A_ receptor levels in reserpine‐treated mice. Previous studies have reported the anti‐inflammatory effects of OFE in various cell lines, such as RAW 264.7 macrophages, rat IEC‐6 small intestine crypt epithelial cells, and human HT‐29 colon epithelial cells. In addition, animal studies have shown the anti‐inflammatory properties of OFE in lung‐injured mice, D‐galactose‐induced cognitive impaired mice, concanavalin A‐induced liver‐injured mice, and brain‐impaired rats (Jing et al., 2015; Lee et al., 2011; Li et al., 2018; Li et al., 2023; Luan et al., 2019; Song et al., 2021; Zhang et al., 2021). However, to the best of our knowledge, the potential correlation between the anti‐inflammatory activity of *O. fragrans* and its ability to alleviate symptoms of depression have not been documented.

Several studies have shown that excessive neuroinflammation is associated with the pathophysiology of depression (Beurel et al., 2020). Based on previous reports, inflammatory mediators are important regulators in the neuroinflammatory mechanism of depression. The overproduction of proinflammatory cytokines promotes the initiation and deterioration of depression (Bauer & Teixeira, 2018). Several studies have reported that patients with depression exhibit significantly elevated levels of pro‐inflammatory cytokines, including TNF‐α, IL‐1β, and IL‐6 (Dowlati et al., 2010). Microglial cells play a crucial role in innate immunity and are responsible for regulating cytokine levels in the CNS (Harry & Kraft, 2008). Upon activation, microglial cells release inflammatory cytokines, such as IL‐6, TNF‐α, and IL‐1β, which are associated with the neuronal degeneration often observed in depression (Fan et al., 2018). Notably, NF‐κB plays a pivotal role in the activation of microglial cells, which in turn leads to the production of cytokines (Chiarugi & Moskowitz, 2003). In the present study, we showed that OFE reduced the production of these proinflammatory cytokines, inhibited microglial cell activation, and suppressed NF‐κB expression. These results suggest that OFE exhibits anti‐inflammatory potential that alleviates neuroinflammation induced by monoamines. To further investigate the direct anti‐inflammatory effects of OFE, we conducted an in vitro experiment using an LPS‐stimulated BV‐2 microglial activation model. The results were consistent with those recorded in reserpine‐treated mice. Treatment with OFE significantly inhibited the production of inflammatory mediators (including TNF‐α, IL‐6, and NO) and suppressed the expression of Iba‐1 and NF‐κB, both of which were stimulated by LPS in BV‐2 microglial cells. Furthermore, a single administration of OFE alleviated LPS‐induced depressive symptoms in mice, suggesting that the anti‐inflammatory properties of OFE may contribute to its antidepressant effects.

In summary, we found that a single administration of OFE significantly reduced the immobility duration in the FST and TST without affecting locomotor activity. Furthermore, OFE exhibited selective enhancing effects on 5‐HTergic function in vivo, as shown by its potentiation effects on 5‐HTP‐induced head‐twitch behavior and its alleviation of reserpine‐induced ptosis deficits. In addition, OFE increased 5‐HT concentration and upregulated 5‐HT_1A_ expression in reserpine‐treated mice, further supporting its effects on 5‐HT transmission. Notably, OFE significantly alleviated microglia activation and the production of inflammatory mediators, both in reserpine‐treated mice in vivo and in LPS‐stimulated BV‐2 cells. These findings suggest that OFE has the potential to be utilized as a dietary supplement for depression by modulating the 5‐HT system and suppressing neuroinflammation.

## AUTHOR CONTRIBUTIONS

Lu‐yao Luo: investigation, writing–original draft. Rui Xue: conceptualization, writing–original draft. Ting‐ge Wang, Jing‐wen Zhang, Shuo Li, Jin‐cao Li, Qiong‐yin Fan, Hua‐jin Dong: investigation. Yang Zhang: conceptualization, writing–original draft. You‐zhi Zhang: writing–review & editing, funding acquisition, supervision.

## FUNDING INFORMATION

None.

## CONFLICT OF INTEREST STATEMENT

The authors declare that they have no conflict of interest.

## ETHICS STATEMENT

This study was approved by the Institutional Animal Care and Use Committee of Beijing Institute of Pharmacology and Toxicology (Ethical No. IACUC‐DWZX‐2021‐609).

## Data Availability

All data and analytical methods in this study are available upon reasonable requests.
